# Editorial: Community series in unveiling the next generation of cancer immunity & immunotherapy in lung cancer, volume II

**DOI:** 10.3389/fimmu.2026.2005017

**Published:** 2026-10-13

**Authors:** Nethaji Muniraj, Mohanraj Sadasivam, Chellappagounder Thangavel

**Affiliations:** 1 Children’s National Hospital, Center for Cancer and Immunology Research, Washington, DC, United States; 2 Division of Immunology, Department of Internal Medicine, University of Iowa Carver College of Medicine, Iowa City, IA, United States; 3 Iowa City VA Health Care System, Iowa City, IA, United States; 4 Center for Translational Medicine, Department of Medicine, Thomas Jefferson University, Philadelphia, PA, United States

**Keywords:** cancer immunity, ecosystem, immunotherapy, lung cancer, tumor microenvironment

## 1 Introduction

Lung cancer is the leading cause of cancer-related deaths worldwide, despite major advances in screening, diagnosis, and treatment. Approximately 85% of lung cancers are non-small cell lung cancers (NSCLC), while the remaining cases are small cell lung cancers (SCLC), which tend to grow and spread more rapidly, leading to poorer outcomes. Over the past two decades, immune checkpoint inhibitors (ICIs), especially those targeting the PD-1/PD-L1 pathway, have significantly changed how lung cancer is treated. These therapies have improved survival in many patients and established immunotherapy as a standard treatment option alongside conventional therapies. However, only few patients had long-lasting responses, while others respond briefly or develop resistance over time. These differences could be due to the complex relationship between tumors and the immune system, highlighting the need to better understand what drives treatment success and resistance. Collectively, the studies assembled in this Research Topic reflect the rapid evolution of lung cancer immunology by integrating mechanistic discoveries, therapeutic innovation, biomarker development, and clinical translation into a comprehensive perspective on the next-generation cancer immunotherapy (**Figure 1**).

**Figure 1:**
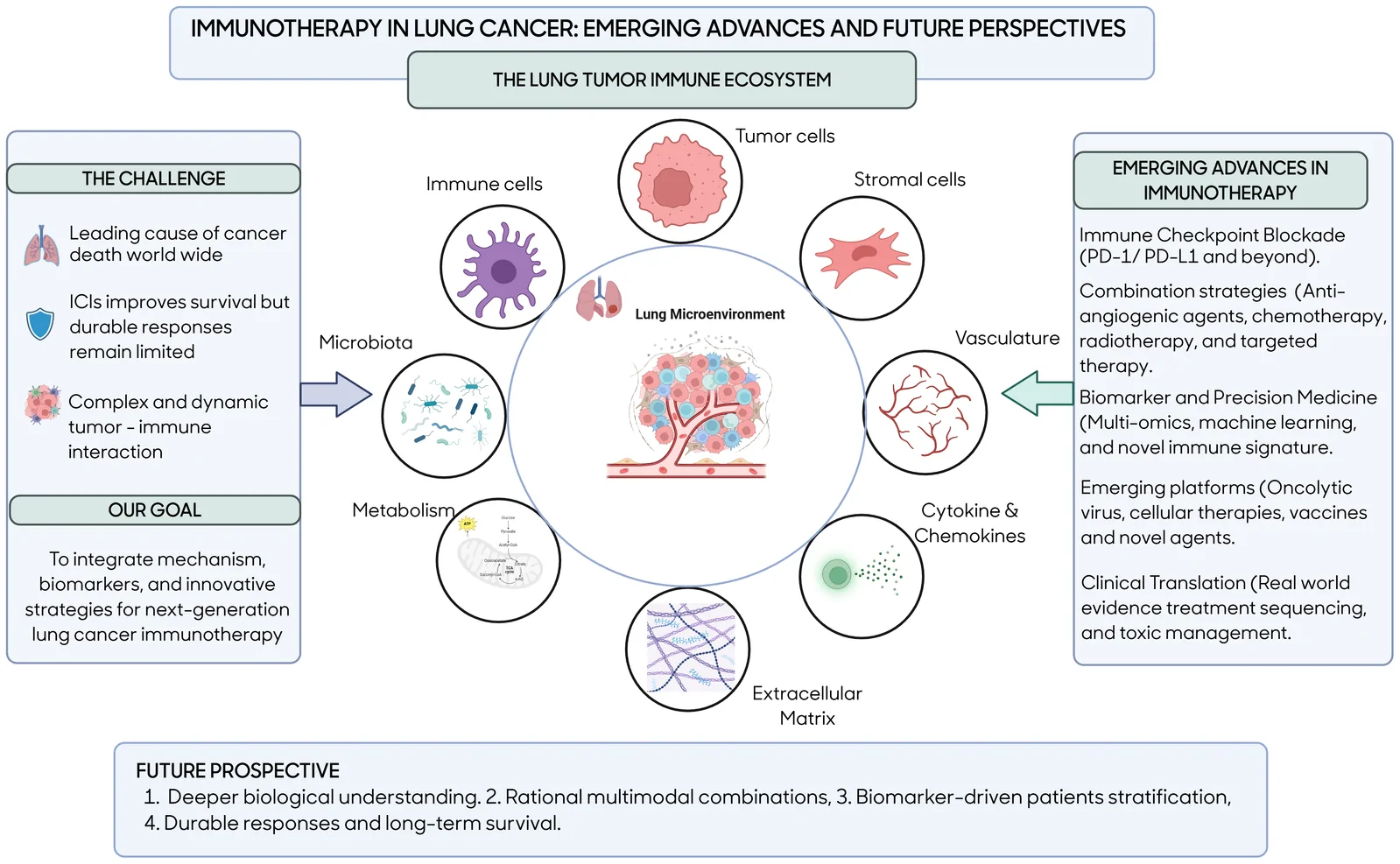
The immune microenvironment of lung cancer is the interaction between tumor cells, immune cells, and stroma cells, vasculature, microbiome, metabolic pathways, extracellular matrix, and cytokines. New approaches for therapy include combinations of immune checkpoint inhibitors with different treatments and biomarker guidance along with new technologies in immunotherapy. The clinical applications of these developments will result in better patient selection and treatment methods. Further research in this direction should target multimodal treatments.

The contemporary view of cancer immunity has expanded far beyond the classical concept of immune surveillance. Lung tumors are now recognized as dynamic ecosystems in which malignant cells continuously interact with immune cell populations, stromal components, vascular networks, metabolic pathways, and resident microbial communities. These interactions collectively shape the tumor immune microenvironment (TIME), influencing immune cell recruitment, activation, exhaustion, and ultimately, therapeutic responsiveness. Consequently, effective immunotherapy is increasingly viewed not simply as the restoration of T-cell function alone, but as the coordinated modulation of an intricate biological network. Recent advances in immunometabolism, angiogenesis, extracellular matrix remodeling, microbiome research, and non-classical immune checkpoints have substantially broadened our understanding of immune escape mechanisms while revealing new therapeutic opportunities. This shift—from targeting isolated immune pathways to comprehensively remodeling the tumor ecosystem—represents one of the defining concepts emerging from this collection.

A parallel transformation is occurring in precision oncology. Although PD-L1 expression and tumor mutational burden have become important biomarkers for guiding immunotherapy, their predictive value remains limited by the remarkable biological heterogeneity of lung cancer. The integration of multi-omics technologies, including genomic, transcriptomic, metabolomic, and single-cell analyses, is providing unprecedented insights into the molecular networks that regulate tumor progression and antitumor immunity. These approaches are enabling the development of increasingly sophisticated prognostic models that integrate molecular and clinical information to improve patient stratification and therapeutic decision-making. At the same time, advances in computational biology and machine learning are accelerating the discovery of novel immune-related biomarkers capable of capturing the dynamic complexity of the tumor ecosystem. Rather than relying on individual biomarkers, future immunotherapeutic strategies are expected to integrate multiple biological dimensions to guide personalized treatment.

Therapeutic innovation has progressed alongside these biological discoveries. Beyond conventional immune checkpoint blockade, rational combination strategies incorporating anti-angiogenic agents, chemotherapy, radiotherapy, and emerging immunotherapeutic platforms seek to overcome primary and acquired resistance by simultaneously targeting complementary mechanisms of immune regulation. Furthermore, clinical studies, prospective protocols, systematic reviews, and case reports of exceptional responders provide valuable evidence supporting the translation of these advances into routine clinical practice. Together, the contributions to this Research Topic illustrate a clear transition from conventional checkpoint inhibition toward ecosystem-based precision immunotherapy, in which therapeutic success depends on integrating mechanistic insights, biomarker-guided patient selection, and multidisciplinary clinical management. In this Editorial, we synthesize these advances and discuss how they collectively define the future direction of cancer immunity and immunotherapy in lung cancer (**Table 1**).

**Table 1:** Overview of the studies included in the Research Topic and their contribution to next-generation lung cancer immunotherapy.

Scientific theme	Representative study	Study type	Major contribution	Reference
Tumor immune ecosystem	Intratumoral microbiota in lung cancer	Review	Highlights the role of intratumoral microbiota in regulating immune surveillance, therapeutic response, and the tumor immune microenvironment (TIME).	Zhang et al.
Immune regulation	CD137/CD137L and CD200/CD200R expression in metastatic lymph nodes	Original Research	Expands understanding of non-classical immune checkpoint pathways involved in immune escape and potential therapeutic targeting.	Kwiecien et al.
Immunometabolism and Precision biomarkers	Energy metabolism-related prognostic signature	Original Research	Identifies metabolism-associated biomarkers linked to prognosis, immune infiltration, and immunotherapy responsiveness.	Xie et al.
Computational oncology	Depression-associated prognostic model using machine learning and single-cell analysis	Original Research	Demonstrates how computational approaches improve biomarker discovery and patient stratification.	Zheng et al.
Anti-angiogenic Combination immunotherapy	PD-1/PD-L1 plus VEGF-targeted therapy	Systematic Review & Meta-analysis	Supports vascular normalization as a strategy to enhance immune checkpoint blockade and improve survival in advanced NSCLC.	Mao et al.
Tumor microenvironment remodeling	ARMOR I clinical trial protocol	Clinical Trial Protocol	Investigates angiotensin receptor blockade combined with chemoimmunotherapy to remodel the tumor microenvironment and improve immune infiltration.	Wang et al.
Radiotherapy–immunotherapy	Radiotherapy combined with immune checkpoint inhibitors	Systematic Review & Meta-analysis	Demonstrates synergistic effects between radiotherapy and immunotherapy through enhanced systemic immune activation.	Wang et al.
Extensive-stage SCLC immunotherapy	Reviews and real-world clinical studies	Review & Real-world Studies	Highlights advances in chemoimmunotherapy, treatment sequencing, continuation of immunotherapy, and individualized management of ES-SCLC.	Xu et al.
Emerging therapeutic platforms	Oncolytic vaccinia virus	Review	Describes vaccinia virus as an emerging immunotherapeutic platform capable of inducing immunogenic cell death and enhancing antitumor immunity.	Li et al.
Clinical translation	Exceptional responder case reports	Case Reports	Illustrate the value of multidisciplinary treatment strategies and personalized therapeutic approaches in achieving durable clinical responses.	Junjun et al.

## 2 Deciphering the lung tumor immune ecosystem: beyond immune checkpoint blockade

The clinical success of immune checkpoint inhibitors has transformed lung cancer therapy, yet the observation that only a subset of patients achieves durable responses underscores the complexity of the tumor immune microenvironment (TIME). Rather than functioning as isolated therapeutic targets, immune checkpoints operate within a dynamic ecosystem comprising malignant cells, immune infiltrates, stromal fibroblasts, endothelial cells, extracellular matrix components, vascular networks, metabolic pathways, and microbial communities. These interconnected elements collectively determine immune surveillance, tumor progression, and responsiveness to therapy. Consequently, understanding the biological complexity of the TIME has become fundamental to the development of next-generation immunotherapeutic strategies.

Several studies in this Research Topic highlight emerging regulators of the lung tumor ecosystem that extend beyond the classical PD-1/PD-L1 axis. Growing evidence indicates that the intratumoral microbiota actively influences immune activation, inflammatory signaling, and therapeutic responsiveness, while non-classical immune checkpoints, including CD137/CD137L and CD200/CD200R, further expand the regulatory networks governing antitumor immunity. In parallel, stromal remodeling and angiogenesis create both physical and immunological barriers that restrict immune cell infiltration and promote therapeutic resistance. These observations support the concept that vascular normalization and stromal modulation are not merely complementary to immunotherapy but integral to restoring effective immune surveillance. Likewise, advances in immunometabolism demonstrate that metabolic reprogramming shapes both tumor cell survival and immune cell function, linking nutrient utilization, hypoxia, and mitochondrial activity to treatment response. Collectively, these findings redefine the TIME as a highly integrated biological ecosystem in which microbial, metabolic, vascular, stromal, and immune signaling networks converge to regulate tumor evolution. Rather than targeting individual pathways in isolation, future therapeutic strategies will likely require the coordinated modulation of multiple components of this ecosystem to achieve durable clinical benefit.

## 3 Next-generation immunotherapeutic strategies: from combination therapies to novel treatment platforms

Advances in our understanding of the TIME have driven a paradigm shift from single-agent immune checkpoint blockade toward rational combination immunotherapy. The studies included in this Research Topic demonstrate that integrating immune checkpoint inhibitors with anti-angiogenic therapy, radiotherapy, chemotherapy, and stromal-modulating approaches can simultaneously address complementary mechanisms of immune resistance while enhancing antitumor immunity. In particular, evidence supporting the combination of PD-1/PD-L1 blockade with VEGF-targeted therapies illustrates how vascular normalization can improve immune cell trafficking and potentiates checkpoint inhibition. Similarly, strategies incorporating angiotensin receptor blockade seek to remodel the stromal microenvironment, whereas radiotherapy is increasingly recognized as an immunological partner capable of inducing immunogenic cell death, enhancing antigen presentation, and promoting systemic immune activation. Together, these studies reinforce the concept that effective immunotherapy increasingly depends on biologically informed combinations that target multiple components of the tumor ecosystem rather than individual immune pathways alone.

This therapeutic evolution is equally evident in extensive-stage small cell lung cancer (ES-SCLC), where real-world studies and comprehensive reviews demonstrate continued progress in optimizing chemoimmunotherapy, treatment sequencing, and patient selection. Emerging evidence support the integration of immune checkpoint inhibitors with anti-angiogenic agents and other multimodal approaches while highlighting the importance of individualized clinical management beyond standardized treatment algorithms. At the same time, innovative platforms such as oncolytic virotherapy exemplify how direct tumor lysis can be coupled with activation of innate and adaptive immunity to convert immunologically “cold” tumors into treatment-responsive lesions. Complemented by exceptional responder case reports and multidisciplinary treatment strategies, these advances collectively emphasize that the future of lung cancer immunotherapy lies in adaptive, ecosystem-based therapeutic approaches that integrate mechanistic understanding with clinical innovation. Rather than relying solely on increasingly potent immunotherapeutic agents, the next generation of treatment will depend on intelligently combining complementary modalities to maximize durable antitumor immunity, while minimizing therapeutic resistance. This evolving therapeutic landscape naturally leads to the next challenge in lung cancer immunology: identifying robust biomarkers capable of guiding precision immunotherapy for individual patients.

## 4 Toward precision immunotherapy: biomarkers, multi-omics, and personalized therapeutic decision-making

Despite the remarkable success of immune checkpoint inhibitors, accurately predicting which patients will achieve durable clinical benefit remains one of the greatest challenges in lung cancer immunotherapy. Although biomarkers such as PD-L1 expression and tumor mutational burden have improved patient selection, their predictive value remains limited because they capture only a fraction of the biological complexity underlying tumor–immune interactions. The studies assembled in this Research Topic collectively demonstrate a shift from reliance on single biomarkers toward integrated precision immunotherapy, in which molecular, immunological, and clinical information is combined to better characterize tumor heterogeneity and therapeutic responsiveness. Multi-omics approaches—including transcriptomic profiling and, immune landscape analyses, together with machine learning–based prognostic models—provide increasingly sophisticated insights into the molecular networks governing immune regulation, disease progression, and treatment outcomes. Likewise, metabolism-related signatures and computational risk models illustrate how systems biology can identify clinically relevant biomarkers associated with immune infiltration, prognosis, and responsiveness to immunotherapy. These advances support a broader vision of precision oncology in which biomarker discovery extends beyond individual molecules to encompass the dynamic interactions among tumor cells, immune populations, metabolism, and the surrounding microenvironment.

Importantly, precision immunotherapy should be viewed as a continuously evolving process rather than as a single diagnostic decision. Dynamic assessment of treatment response, immune remodeling, and metabolic adaptation may ultimately provide greater clinical value than static baseline measurements alone. The integration of artificial intelligence, computational pathology, single-cell technologies, and longitudinal molecular profiling offers unprecedented opportunities to refine patient stratification and optimize therapeutic sequencing throughout disease progression. Collectively, the studies presented in this Research Topic reinforce the concept that future therapeutic decisions will be guided by comprehensive biological profiling of the tumor ecosystem, enabling more individualized and adaptive treatment strategies that bridge mechanistic discovery with clinical implementation.

## 5 From discovery to clinical translation: challenges and future perspectives

The contributions within this Research Topic clearly demonstrate that the next generation of lung cancer immunotherapy will depend not only on scientific discovery but also on the successful translation of these advances into routine clinical practice. Despite substantial progress in understanding the tumor immune microenvironment, developing combination immunotherapies, and identifying novel biomarkers, therapeutic resistance, tumor heterogeneity, and dynamic immune adaptation continue to limit durable clinical responses. Overcoming these barriers will require greater integration of mechanistic biology with prospective clinical trials, real-world evidence, and multidisciplinary patient management. Rather than evaluating therapeutic strategies in isolation, future research should emphasize rational treatment sequencing, biomarker-guided patient selection, and adaptive therapeutic approaches capable of responding to the evolving biological characteristics of individual tumors.

Emerging technologies are expected to accelerate this transition toward precision oncology. Single-cell and spatial multi-omics, longitudinal immune monitoring, computational pathology, and artificial intelligence are transforming our ability to characterize the temporal and spatial complexity of tumor–immune interactions. At the same time, innovative therapeutic platforms—including oncolytic virotherapy, engineered cellular therapies, novel immune checkpoint modulators, and rational multimodal treatment strategies—continue to expand the therapeutic landscape beyond conventional checkpoint inhibition. The integration of these technologies with molecular profiling, clinical expertise, and real-world data has the potential to generate truly personalized therapeutic strategies that continuously adapt throughout the course of disease. Ultimately, the studies highlighted in this Research Topic demonstrate that the next generation of lung cancer immunotherapy will emerge through the convergence of systems biology, translational research, computational medicine, and multidisciplinary clinical collaboration, providing a comprehensive framework for individualized cancer care and improved long-term patient outcomes.

## 6 Conclusion

The studies presented in the *Community Series in Unveiling the Next Generation of Cancer Immunity & Immunotherapy in Lung Cancer: Volume II* collectively demonstrate the remarkable evolution of lung cancer immunology from mechanistic discovery toward precision, patient-centered immunotherapy. Together, they illustrate that effective cancer immunotherapy extends far beyond immune checkpoint blockade and increasingly depends on understanding the complex interplay among tumor cells, immune populations, stromal architecture, angiogenesis, metabolism, microbiota, and the broader tumor immune ecosystem. By integrating fundamental biology with translational research, clinical investigations, and real-world evidence, this collection highlights how complementary scientific disciplines are converging to improve patient stratification and therapeutic outcomes.

A central message emerging from this Research Topic is that the future of lung cancer immunotherapy will be driven not by incremental improvements in individual therapeutic agents, but by the integration of biologically informed combination therapies, multidimensional biomarker discovery, and adaptive precision medicine. Advances in multi-omics technologies, artificial intelligence, computational biology, and longitudinal immune profiling provide increasingly sophisticated tools for characterizing tumor heterogeneity and guiding individualized therapeutic decisions. Equally important, the continued integration of laboratory discoveries with prospective clinical trials, real-world evidence, and multidisciplinary collaboration will be essential for translating scientific innovation into durable clinical benefit.

Collectively, the contributions summarized in this editorial reinforce a paradigm shift toward ecosystem-based precision immunotherapy, in which treatment decisions are informed by comprehensive molecular, immunological, and clinical profiling rather than isolated biomarkers or single therapeutic modalities. As our understanding of the tumor immune ecosystem continues to expand, integrating biological insight with therapeutic innovation and clinical implementation will be fundamental to overcoming treatment resistance and improving long-term outcomes. This evolving framework provides a strong foundation for the next generation of personalized immunotherapeutic strategies and offers renewed optimism for advancing the care of patients with lung cancer.

